# Comparison of 3-factor versus 4-factor prothrombin complex concentrate for emergent warfarin reversal: a systematic review and meta-analysis

**DOI:** 10.1186/s12873-022-00568-x

**Published:** 2022-01-24

**Authors:** David J. Margraf, Sarah J. Brown, Heather L. Blue, Tamara L. Bezdicek, Julian Wolfson, Scott A. Chapman

**Affiliations:** 1 Department of Experimental and Clinical Pharmacology, College of Pharmacy, University of Minnesota, 7-115E Weaver-Densford Hall, 308 Harvard St. SE, Minneapolis, MN 55455 USA; 2 Health Sciences Libraries, University of Minnesota, Minneapolis, MN USA; 3 Department of Pharmacy Practice and Pharmaceutical Sciences, College of Pharmacy, University of Minnesota, Duluth, MN USA; 4 Fairview Pharmacy Services, Minneapolis, MN USA; 5 Division of Biostatistics, School of Public Health, University of Minnesota, Minneapolis, MN USA

**Keywords:** Blood coagulation factors, Warfarin, Meta-analysis, Prothrombin complex concentrate, Systematic review

## Abstract

**Background:**

Patients requiring emergent warfarin reversal (EWR) have been prescribed three-factor prothrombin complex concentrate (PCC3) and four-factor prothrombin complex concentrate (PCC4) to reverse the anticoagulant effects of warfarin. There is no existing systematic review and meta-analysis of studies directly comparing PCC3 and PCC4.

**Methods:**

The primary objective of this systematic review and meta-analysis was to determine the effectiveness of achieving study defined target INR goal after PCC3 or PCC4 administration. Secondary objectives were to determine the difference in safety endpoints, thromboembolic events (TE), and survival during the patients’ hospital stay. Random-effects meta-analysis models were used to estimate the odds ratios (OR), and heterogeneity associated with the outcomes. The Newcastle-Ottawa Scale was used to assess study quality, and Preferred Reporting Items for Systematic Reviews and Meta-Analyses (PRISMA) guidelines were followed.

**Results:**

Ten full-text manuscripts and five abstracts provided data for the primary and secondary outcomes. Patients requiring EWR had more than three times the odds of reversal to goal INR when they were given PCC4 compared to PCC3 (OR = 3.61, 95% CI: 1.97–6.60, *p* < 0.001). There was no meaningful clinical association or statistically significant result between PCC4 and PCC3 groups in TE (OR = 1.56, 95% CI: 0.83–2.91, *p* = 0.17), or survival during hospital stay (OR = 1.34, 95% CI: 0.81–2.23, *p* = 0.25).

**Conclusion:**

PCC4 is more effective than PCC3 in meeting specific predefined INR goals and has similar safety profiles in patients requiring emergent reversal of the anticoagulant effects of warfarin.

**Supplementary Information:**

The online version contains supplementary material available at 10.1186/s12873-022-00568-x.

## Background

Critical bleeding associated with warfarin anticoagulation necessitates rapid reversal and return of normal hemostasis to halt the progression of bleeding and facilitate emergent surgery. Fresh frozen plasma (FFP), vitamin K, and prothrombin complex concentrates (PCC) are recommended therapies for INR correction [1–8].

Several activated and non-activated PCC products are available and have been administered to patients for the reversal of warfarin anticoagulation [1]. These products are lyophilized plasma-derived concentrates used intravenously after reconstitution that differ in their coagulation factor components. While all non-activated PCC products contain factors II, IX, and X, they differ in their amount of factor VII, with three-factor PCC (PCC3) products providing low concentrations of factor VII relative to the other factors while four-factor PCC (PCC4) products contain higher concentrations of factor VII. Additionally, PCC products differ by other components of clinical concern. Kcentra®, the PCC4 product available in the United States (US), contains anticoagulant proteins C and S, heparin, antithrombin III, and human albumin [9]. Bebulin® VH, a now-discontinued PCC3 product, contained heparin, whereas Profilnine® SD, another PCC3 product, does not contain heparin [10, 11]. Heparin is a concern for patients with previous heparin-induced thrombocytopenia (HIT). Other factor-containing products, such as activated protein complex concentrate (FEIBA®) and recombinant factor VIIa (NovoSeven®) have also been evaluated for their effectiveness in reversing INR in emergent warfarin reversal (EWR) [12–15].

Before 2013, only PCC3 products were available in the US. These products were administered for EWR off-label, and with no specific dosing guidance available. A clinical trial evaluating PCC4 in comparison to FFP using a specified dosing regimen for warfarin reversal with the endpoint of INR correction led to FDA approval of the blood product and is indicated for urgent reversal of acquired coagulation factor deficiency induced by vitamin K antagonist (VKA, e.g., warfarin) therapy in adult patients with acute major bleeding or the need for urgent surgery or an invasive procedure [9, 16, 17]..

Both PCC3 and PCC4 products have been evaluated for their effectiveness in INR correction in EWR, and both reduce the INR more rapidly than plasma [18]. While there have been studies comparing the INR lowering effects of these products, it remains unclear if there is a difference in the measured effectiveness and safety parameters of PCC3 and PCC4 in terms of reliable and predictable INR lowering response to a goal INR, any thromboembolic events (TE), or mortality. The guidelines recommending PCC4 over PCC3 for warfarin anticoagulation reversal are largely the result of the FDA approval of a PCC4 product with an indication for EWR and its associated clinical trials [1]. No prospective comparison of safety and efficacy or patient outcome has been conducted to support the recommendation of PCC4 over PCC3.

Since no randomized control trials have compared PCC3 to PCC4, clinicians are left with observational comparisons and theory based on differing factor VII content as a basis of choosing one PCC product over another. One systematic review without meta-analysis compared studies investigating PCC3 or PCC4 from various institutions [19]. Since its publication, several direct comparisons have been published as retrospective and observational studies [20–34].

This systematic review and meta-analysis of studies directly compares PCC3 to PCC4 in adult patients needing warfarin reversal for bleeding, surgical intervention, or trauma in patients taking warfarin before their hospital admission.

## Methods

The Preferred Reporting Items for Systematic Reviews and Meta-Analyses (PRISMA) guidelines were followed to create our study protocol and conduct the review and analysis [35]. A pharmacy librarian (SB) created the literature search strategy after meeting with two members of the research team (DM, SC) to clarify goals and further define selection criteria. The database search strategy was built and tested for sensitivity in Ovid MEDLINE using medical subject headings (see Additional file 1: Appendix A), and the search strategy was translated to three other databases: Cochrane Library, EMBASE, and Scopus. Databases were chosen to be inclusive of international medical and pharmaceutical literature. References of included studies were also hand searched. The electronic literature searches spanned from database inception to August 20th, 2020, and were executed without limits on date or language.

### Study selection

Studies were included if they met the following criteria: 1) Adult patients needing EWR for bleeding, need for emergent surgical intervention, or trauma patients receiving warfarin anticoagulation before hospital admission; 2) A measured baseline or initial INR was obtained before PCC administration; 3) A PCC dose was administered; 4) At least one INR was obtained after PCC administration; 5) The study design fell into one of the following categories: randomized controlled trial (RCT); prospective or retrospective cohort study; case-control study; 6) The study compared PCC3 versus PCC4 at the same institution; 7) The study was published in English.

Studies were excluded if 1) children or animals were included subjects; 2) The study protocol required a PCC product and concomitant activated recombinant factor VII (rFVIIa, NovoSeven®) and/or activated prothrombin complex concentrates (aPCC; FEIBA®); 3) included patients with hemophilia; 4) The study design fell into one of the following categories: cross-sectional study; case reports; case series. Note that the use of rFVIIa and/or aPCC in some patients did not meet our exclusion criterion.

### Data collection

Duplicate references were removed and items were uploaded to Rayyan [36] for independent screening by two study authors (DM, SC). Screeners met to discuss and resolve conflicts by consensus. Titles were included in the full-text screening when consensus could not be met. The selected studies were uploaded for full-text screening and independent review by two study authors (DM, SC) for selection. Data collection was carried out independently by four study authors (DM, SC, HB, and TB) using a customized data extraction form after piloting the form with a similar study. Manuscripts were assigned and reviewed by random pairs of reviewers. Any conflicts were resolved by the pairs. The data collected from the articles included: title, publication type, the country in which the study was conducted, funding source and role of funders, possible conflicts of interest, type of study, special population characteristics, indication for warfarin reversal, number of study sites, study start and end date, institutional dosing range or strategy, maximum reported INR value by study lab, number of patients in total and by group, age weight, sex, bleeding type, INR collection method, outcome definitions, desired post-PCC INR goal, goal achievement, statistical methods, INR change, thromboembolism screening method and number of TE, survival or death outcomes reported, PCC dose, the time between initial INR and PCC dose given, the time between the PCC dose and the second INR, initial/baseline INR, post-PCC INR, vitamin K usage, FFP usage, the study defined strength, limitations, strategies to overcome the limitations, and key conclusions of study authors. If a study reported patient outcomes for other anticoagulants, (e.g. rivaroxaban), we extracted the relevant warfarin-related data when possible.

### Data analysis

Data from the individual studies were compared qualitatively for clinical importance and relevance before statistical analysis. Measures of central tendency (mean and median) for continuous variables, their associated standard deviations, interquartile ranges, and minimum to maximum ranges are presented in the table and discussed for clinical relevance to avoid bias that would occur by transforming and combining values [37]. The study-specific odds ratios (OR) comparing the two PCC types were calculated using the proportion of patients achieving the study-specific INR goal, the reported number of thromboembolic events, and the reported number of patients that survived during the hospital stay. Random-effects models (REM) were used to estimate the average ORs for each outcome, and the corresponding 95% confidence intervals (CI). Heterogeneity, τ^2^, was estimated, which is the between-study variance of the individual study ORs [38]. Cochran’s *Q*-test, which uses a chi-square distribution, was performed to test whether heterogeneity, τ^2^, equals zero [39]. The *I*^2^-statistic was also calculated, which estimates the percent of the total variation due to heterogeneity [40, 41].

The models were then stratified by publication type: abstract or journal article. Fixed-effects models were not explored because REMs account for the variation in characteristics such as different populations and study structures [42]. Overviews of the individual study results and OR estimates based on the meta-analysis models are displayed graphically with forest plots [43]. Evidence of outliers and influential studies in the model were examined with studentized residuals and Cook’s distances [44]. Studies with zero outcome events in a PCC group had the value of 0.5 added to aid in the calculation of the OR [41]. Given the debate on how informative studies are about treatment effects when both treatment arms have zero outcome events [41], authors analyzed the outcome with and without these studies to see if there was a major impact on the interpretation of the results. Studies reporting summary outcome statistics without reporting the number of patients in each group were included in the results and discussion but excluded from the meta-analysis models.

We included all information found during the systematic review search, including abstracts that were not peer-reviewed. However, since abstracts often do not provide enough detailed information to extract estimates of treatment effect, and it is common to find discordance between abstracts and their corresponding full-text publications [45], we stratified the meta-analysis outcomes by peer-reviewed manuscripts and abstracts. Study quality and the risk of bias were assessed using a Newcastle-Ottawa Scale (NOS) modified to our study needs [46]. Publications with a score of 8 were considered at low risk of bias, 6 or 7 at moderate risk, and 5 or less at high risk. The NOS scale questions are available in Additional file 1: Appendix B.

Publication bias**,** which is created by underreporting of studies by authors that fail to find a positive association, preferable *p*-value, or selective reporting within studies, can affect the cumulative evidence a meta-analysis attempts to provide and can threaten the validity of a meta-analysis. This risk of bias was assessed with funnel plots that were inspected visually and checked for asymmetry, which indicates publication bias, using the rank correlation test [47] and the regression test [48] with the standard error of the observed outcomes as a predictor.

### Additional analyses

To ensure the findings were not heavily impacted by any study, leave-one-out sensitivity analyses were performed by removing one study at a time from the REMs [41]. In addition to the OR estimates obtained from the REMs, conditional logistic mixed-effects models with exact likelihood based on generalized linear mixed-models (GLMM) with a logit link function were explored to see if the OR estimates from a theoretically appropriate analysis [42] differ from the results provided by the REM commonly used in meta-analysis. All analysis and plots were generated with *R* (version 4.0.2) [49] and the *metafor* package (version 2.4.0) [41].

## Results

### Study characteristics

**Fig. 1:**
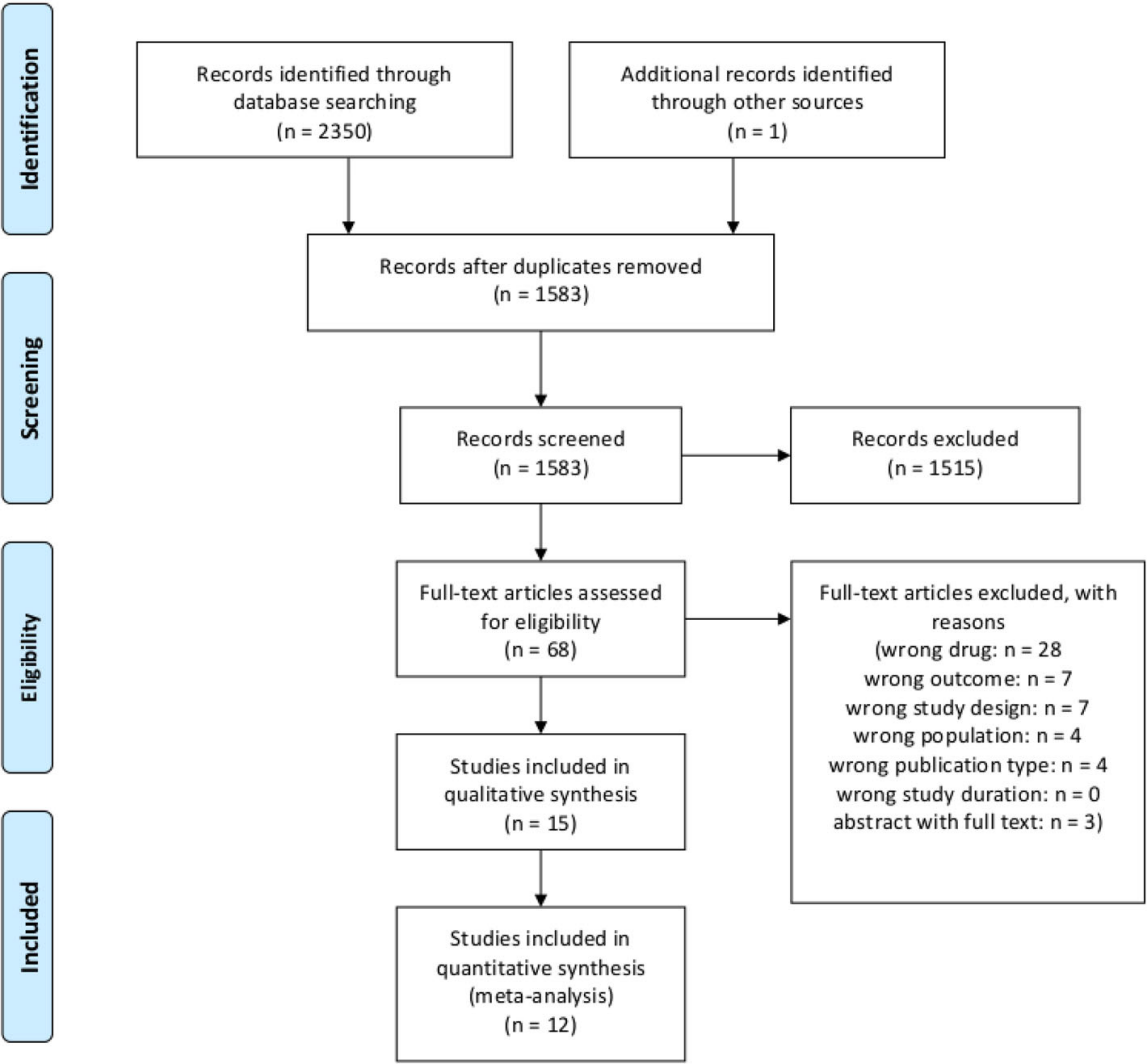
PRISMA flow diagram

Our search strategy identified 1583 studies for screening after duplicates were removed, including results from our previous research [31]. Initial screening in Rayyan [36] resulted in 68 studies uploaded for full-text screening and independent review. As to not use results from two publications on the same study population, three abstracts with subsequent full-text publications were removed [50–52]. Of the remainder, 15 were selected based on inclusion and exclusion criteria for the qualitative synthesis (systematic review). Three of these 15 were excluded [21, 29, 34], leaving 12 included [20, 22–28, 30–33] for the quantitative synthesis (meta-analysis). A PRISMA flow diagram of study inclusion is shown in Fig. 1.

Summary characteristics, quality scores and assessments, and demographics of the included studies are provided in Tables 1 and 2. All studies were retrospective cohort studies providing outcomes of patients treated with PCC3 or PCC4 from 2007 to 2015. One manuscript evaluated patients who received PCC for rivaroxaban reversal as well as warfarin reversal [30]. Reversal to INR goal was limited to the warfarin group in this study. Another study was primarily concerned with non-warfarin-related coagulopathy; however, the results from patients on warfarin reversed with PCCs were still extracted [32].

**Table 1:** Study characteristics

Study, Year	Publication Type	Study Design	Study Sites (***n***)	Patients (***n***)	Quality Score	Risk of Bias Assessment
Al-Majzoub et al., 2016 [20]	Manuscript	Retrospective cohort	1	53	6	Moderate
DeAngelo et al., 2018 [22]	Manuscript	Retrospective cohort	2	89	6	Moderate
Fischer et al., 2018 [24]	Manuscript	Retrospective cohort	22	103	6	Moderate
Holt et al., 2018 [25]	Manuscript	Retrospective cohort	5	134	6	Moderate
Jones et al., 2016 [27]	Manuscript	Retrospective cohort	4	148	6	Moderate
Kuroski et al., 2017 [28]	Manuscript	Retrospective cohort	1	137	6	Moderate
Mangram et al., 2016 [30]^a^	Manuscript	Retrospective cohort	2	61*	6	Moderate
Margraf et al., 2020 [31]	Manuscript	Retrospective cohort	1	80	–	–
Mohan et al., 2018 [32]	Manuscript	Retrospective cohort	2	128	6	Moderate
Voils et al., 2015 [33]	Manuscript	Retrospective cohort	1	165	6	Moderate
Cang et al., 2014 [21]	Abstract	Retrospective cohort	1	NR	4	High
Di Napoli et al., 2014 [23]	Abstract	Retrospective cohort	3	69	5	High
Kotsianas et al., 2015 [26]	Abstract	Retrospective cohort	3	91	6	Moderate
Peck et al., 2016 [29]	Abstract	Retrospective cohort	1	89	5	High
Wagner et al., 2019 [34]	Abstract	Retrospective cohort	(many)	250	5	High

^a^ Most of this study characteristics include data from three rivaroxaban patients

We did not do the quality assessment of our previous work marked as “--”

**Table 2:** Demographics

Study, Year	Age PCC3	Age PCC4	Male PCC3 (%)	Male PCC4 (%)	Weight PCC3 (kg)	Weight PCC4 (kg)
Al-Majzoub et al., 2016 [20]	79 [70–87]	82 [76–88]	19 (54.3%)	10 (55.5%)	81.3 (15.0)	74.5 (12.7)
DeAngelo et al., 2018 [22]	68.9 (14.2)	70.0 (13.7)	38 (66.7%)	14 (40.6%)	89.3 (24.9)	80.8 (29.1)
Fischer et al., 2018 [24]	77 [73–82]	80 [73–86]	17 (42.5%)	34 (54%)	NR	NR
Holt et al., 2018 [25]	74 (10.8)	57 (14.7)	43 (55.8%)	29 (50.9%)	87.9 (26.9)	84.9 (22.6)
Jones et al., 2016 [27]	75.5 [64.0–83.0]	72.5 [58.5–80.0]	45 (53.6%)	38 (59.4%)	84.8 [70.2–100.8]	82.5 [72.4–100.3]
Kuroski et al., 2017 [28]	74.5 (NR)	76.2 (NR)	36 (53.0%)	37 (53.6%)	78.7 (NR)	85.7 (NR)
Mangram et al., 2016 [30]	76 (13)	77 (8)	25 (54.3%)	10 (55.5%)	80 (22)	83 (18)
Margraf et al., 2020 [31]	74.0 [62.0–80.0]	66.0 [57.0–82.0]	36 (63.2%)	12 (52.2%)	81.4 [72.1–94.4]	77.8 [64.7–97.8]
Mohan et al., 2018 [32]	73.54 (28–102, 76.50) Mean (range, median) (both groups)	73.54 (28–102, 76.50) Mean (range, median) (both groups)	83 (65%) (both groups)	83 (65%) (both groups)	77.9 (NR) (both groups)	77.9 (NR) (both groups)
Voils et al., 2015 [33]	71.8 (13.1)	70.4 (13.4)	31 (55%)	64 (55%)	85 (25)	88 (23)
Cang et al., 2014 [21]	NR	NR	NR	NR	NR	NR
Di Napoli et al., 2014 [23]	NR	NR	NR	NR	NR	NR
Kotsianas et al., 2015 [26]	NR	NR	NR	NR	NR	NR
Peck et al., 2016 [29]	NR	NR	NR	NR	NR	NR
Wagner et al., 2019 [34]	68 [59, 80] (both groups)	68 [59, 80] (both groups)	NR	NR	NR	NR

Age and weight reported as mean (SD), median [IQR], or mean (min-max, median)

*PCC* prothrombin complex concentrate, *PCC3* 3 factor PCC, *PCC4* 4 factor PCC

The patients in the included studies received either PCC3 or PCC4 for surgery, intracranial hemorrhage, gastrointestinal, or other bleeding types. Nine full-text manuscripts reported giving Kcentra® as the PCC4 product, while the use of PCC3 products varied (Bebulin® VH: *n* = 4, Profilnine® SD: *n* = 5). No abstracts reported the product by proprietary name. The reported weight-based PCC dose (Table 3) was similar for most studies, however, there were some large differences between PCC product dosing in Holt et al. [mean units/kg (SD), PCC3: 24.6 (9.3) vs. PCC4: 36.3 (12.8)], and Mohan, et al. [mean units/kg (SD), PCC3: 40.9 (18) vs. PCC4: 32.2 (11.07)]. The individual studies reported variable initial INR, change in INR, post-PCC INR measurements (Table 4), the timing of PCC administration (Table 5), and bleed type (Table 6). Patients were often given other concomitant agents to reverse INR. Vitamin K was commonly used in both PCC groups as part of the hemostasis protocol in many hospitals. Many studies reported an increased percentage of patients receiving vitamin K with PCC4 than PCC3. The use of FFP was commonly reported, particularly in PCC3 patients.

**Table 3:** Prothrombin complex concentrate brand and first dose

Study, Year	PCC3 brand	PCC4 brand *	Dose PCC3 (u/kg)	Dose PCC4 (u/kg)	Dose PCC3 (units)	Dose PCC4 (units)
Al-Majzoub et al., 2016 [20]	Profilnine® SD	KCentra®	25.5 (4.3)	27.9 (6.9)	NR	NR
DeAngelo et al., 2018 [22]	Profilnine® SD	KCentra®	25 [23–27]	23 [20–27]	2080 [1940–2500]*	1620 [1301–2213]*
Fischer et al., 2018 [24]	Profilnine® SD	KCentra®	26 [20–41]	25 [23–29]	2000 [1500–3248]	2088 [1665–2500]
Holt et al., 2018 [25]	NR	NR	24.6 (9.3)	36.3 (12.8)	NR	NR
Jones et al., 2016 [27]	Bebulin® VH	KCentra®	30.6 [28.2–32.3]	26.3 [24.7–34.3]	2454 [2228–3045]	2500 [2000–2852]
Kuroski et al., 2017 [28]	Bebulin® VH	KCentra®	28.9 (22.5–40.1), median (range)	25 (12–50)	NR	NR
Mangram et al., 2016 [30]	Bebulin® VH	KCentra®	29 (9)	26 (6)	NR	NR
Margraf et al., 2020 [31]	Profilnine® SD	KCentra®	21.5 [20.4–25.9]	29.3 [25.9–37.3]	2000 [1530–2500]	2595 [1880–3307]
Mohan et al., 2018 [32]	Bebulin® VH	KCentra®	40.99 (18), median (40.04)	32.22 (11.07), median (27.35)	3073 (1654)	2472 (930)
Voils et al., 2015 [33]	Profilnine® SD	KCentra®	28 [25–31]	27 [24–31]	2250 [1980–2970]	2250 [1788–2940]
Cang et al., 2014 [21]	NR	NR	NR	NR	NR	NR
Di Napoli et al., 2014 [23]	NR	NR	NR	NR	NR	NR
Kotsianas et al., 2015 [26]	NR	NR	NR	NR	NR	NR
Peck et al., 2016 [29]	NR	NR	NR	NR	NR	NR
Wagner et al., 2019 [34]	NR	NR	NR	NR	NR	NR
		* All US studies are assumed to have used Kcentra®				*First dose

*INR* international normalized ratio, *NR* not reported, *PCC* prothrombin complex concentrate, *PCC3* 3 factor PCC, *PCC4* 4 factor PCC

**Table 4:** INR change, initial/baseline INR, and INR post-PCC

Study, Year	INR change PCC3	INR change PCC4	Initial/baseline INR in PCC3 group	Post-PCC dose INR in the PCC3 group	Initial/baseline INR in PCC4 group	Post-PCC dose INR in the PCC4 group
Al-Majzoub et al., 2016 [20]	0.9 (0.5)	1.8 (1.5)	2.3 (0.6), NR [NR]	1.4 (0.2), NR [NR]	3.0 (1.5), NR [NR]	1.2 (0.1), NR [NR]
DeAngelo et al., 2018 [22]	NR	NR	NR (NR), 2.6 [2.2–3.7]	NR	NR (NR), 2.6 [2.0–3.4]	NR
Fischer et al., 2018 [24]	NR	NR	NR (NR), 2.8 [2.3–3.7]	NR (NR), 1.3 [1.2–1.5]	NR (NR), 2.6 [2.2–3.1]	NR (NR), 1.2 [1.2–1.4]
Holt et al., 2018 [25]	NR	NR	3.61 (2.3), NR [NR]	1.40 (0.27), NR [NR]	6.87 (2.3), NR [NR]	1.25 (0.33), NR [NR]
Jones et al., 2016 [27]	NR	NR	NR (NR), 2.6 [2.2–3.5]	NR (NR), 1.3 [1.2–1.4]	NR (NR), 3.0 [2.2–4.6]	NR (NR), 1.2 [1.1–1.4]
Kuroski et al., 2017 [28]	NR	NR	NR (NR), 3.15 (1.6–19) range	NR (NR), 1.4 [1.1–2.6]	NR (NR), 3.1 (2–19) range	NR (NR), 1.3 [1–1.8]
Mangram et al., 2016 [30]	NR	NR	3.1 (2.3), NR [NR]	1.6 (0.6), NR [NR]	3.4 (3.7), NR [NR]	1.3 (0.2), NR [NR]
Margraf et al., 2020 [31]	1.1 [0.6–2.0]	2.3 [1.2–3.3]	2.8 [2.1–4.1]	1.7 [1.5–2.0]	3.7 [2.6–4.9]	1.3 [1.3–1.4]
Mohan et al., 2018 [32]	2.80 (2.33) (median = 2.13)	3.23 (3.48) (median = 1.85)	4.64 (2.88), 3.72 [NR]	1.85 (0.92), 1.50 [NR]	4.54 (3.45), 3.05 [NR]	1.30 (0.20), 1.3 [NR]
Voils et al., 2015 [33]	1.4 (NR)	2.2 (NR)	3.0 (NR), 2.5 [2.0–3.2]	1.6 (NR)	3.5 (NR), 2.4 [2.0–4.2]	1.3 (NR)
Cang et al., 2014 [21]	NR	NR	NR	NR	NR	NR
Di Napoli et al., 2014 [23]	NR	NR	NR	NR	NR	NR
Kotsianas et al., 2015 [26]	NR	NR	3.44 (1.99)	NR	3.86 (2.50)	NR
Peck et al., 2016 [29]	1.26 (1.11)	3.6 (4.16)	NR	NR	NR	NR
Wagner et al., 2019 [34]	NR	NR	NR	1.5	NR	1.4

mean (SD), median [IQR]

*INR* international normalized ratio, *NR* not reported, *PCC* prothrombin complex concentrate, *PCC3* 3 factor PCC, *PCC4* 4 factor PCC

**Table 5:** Timing of PCC administration

Study, Year	Time between initial INR and PCC dose PCC3	Time between initial INR and PCC dose PCC4	Time between PCC and second INR PCC3	Time between PCC dose and second INR PCC4
Al-Majzoub et al., 2016 [20]	NR	NR	5.0 (7.4) hours	3.7 (4) hours
DeAngelo et al., 2018 [22]	NR	NR	NR (88%)	NR (97%)
Fischer et al., 2018 [24]	NR	NR	NR	NR
Holt et al., 2018 [25]	NR	NR	3.8 (0.12) hours	3.3 (0.10) hours
Jones et al., 2016 [27]	NR	NR	48:59 [31:00–91:00] min:sec	23:40 [15:33–90:00] min:sec
Kuroski et al., 2017 [28]	37.9 (28.3) minutes	42.7 (27) minutes	191 (195) minutes	169 (230) minutes
Mangram et al., 2016 [30]	NR	NR	3 [0.6–16.5] hours	4.2 [0.6–18.9] hours
Margraf et al., 2020 [31]	78 [56.0–113.0] minutes	73 [40.0–108.5] minutes	93 [46.0–228.0] minutes	226 [156.5–368.5] minutes
Mohan et al., 2018 [32]	NR	NR	3 h (both groups)	3 h (both groups)
Voils et al., 2015 [33]	NR	NR	NR	NR
Cang et al., 2014 [21]	NR	NR	NR	NR
Di Napoli et al., 2014 [23]	NR	NR	NR	NR
Kotsianas et al., 2015 [26]	NR	NR	217 (247) minutes	208 (187) minutes
Peck et al., 2016 [29]	NR	NR	NR	NR
Wagner et al., 2019 [34]	NR	NR	NR	NR

*INR* international normalized ratio, *NR* not reported, *PCC* prothrombin complex concentrate, *PCC3* 3 factor PCC, *PCC4* 4 factor PCC

**Table 6:** Patients stratified by bleed type

Study, Year	ICH PCC3	ICH PCC4	GIB PCC3	GIB PCC4	Other PCC3	Other PCC4	Not listed PCC3	Not listed PCC4
Al-Majzoub et al., 2016 [20]	26 (74.3%)	12 (66.7%)	7 (20%)	2 (11.1%)	2 (5.1%)	3 (16.7%)	NR	NR
DeAngelo et al., 2018 [22]	21 (61.8%)	10 (76.9%)	0 (0%)	2 (15.4%)	2 (5.9%)	1 (7.7%)	Thoracic 2 (5.9%), genitourinary GU 1 (2.9%), intraabdominal and retroperitoneal 8 (23.5%)	0 (0%)
Fischer et al., 2018 [24]	100%	100%	0 (0%)	0 (0%)	0 (0%)	0 (0%)	0 (0%)	0 (0%)
Holt et al., 2018 [25]	48 (62%)	23 (40%)	5 (7%)	14 (24%)	13 (16%)	4 (7%)	Chest Bleeds = 4 (6%) Multiple = 7 (9%) Missing = 0	Chest Bleeds PCC4 = 0 Multiple = 15 (25%) Missing = 1 (2%)
Jones et al., 2016 [27]	80 (95.2%)	40 (62.5%)	2 (2.4%)	11 (17.2%)	2 (2.4%)	13 (20.3%)	NR	NR
Kuroski et al., 2017 [28]	48 (70.6%)	54 (78.3%)	4 (5.9%)	2 (2.9%)	12 (17.6%)	10 (14.5%)	Retroperitoneal: 2 (2.9%), Emergent Surgery: 2 (2.9%)	Retroperitoneal: 2 (2.9%), Emergent Surgery: 1 (1.4%)
Mangram et al., 2016 [30]*	NR	NR	NR	NR	NR	NR	NR	NR
Margraf et al., 2020 [31]	31 (54.4%)	19 (82.6%)	10 (17.5%)	2 (8.7%)	16 (28.1%)	2 (8.7%)	NR	NR
Mohan et al., 2018 [32]	45 (35%) (both groups)	45 (35%) (both groups)	33 (26%) (both groups)	33 (26%) (both groups)	29 (23%) (both groups)	29 (23%) (both groups)	Periprocedural 21 (16%) (both groups)	Periprocedural 21 (16%) (both groups)
Voils et al., 2015 [33]	65 (60%)	34 (61%)	7 (6%)	10 (18%)	6 (6%)	0 (0%)	IA/thoracic 12 (11%)	IA/thoracic 8 (14%)
Cang et al., 2014 [21]	NR	NR	NR	NR	NR	NR	NR	NR
Di Napoli et al., 2014 [23]	51 (100%)	18 (100%)	0 (0%)	0 (0%)	0 (0%)	0 (0%)	0 (0%)	NR
Kotsianas et al., 2015 [26]	100%	100%	0 (0%)	0 (0%)	0 (0%)	0 (0%)	0 (0%)	0 (0%)
Peck et al., 2016 [29]	100%	100%	0 (0%)	0 (0%)	0 (0%)	0 (0%)	0 (0%)	0 (0%)
Wagner et al., 2019 [34]	62%	50%	NR	NR	NR	NR	NR	NR

*GIB* gastrointestinal bleeding, *ICH* intracerebral hemorrhage, *INR* international normalized ratio, *NR* not reported, *PCC* prothrombin complex concentrate, *PCC3* 3 factor PCC, *PCC4* 4 factor PCC

### Achieving INR goal

Eight full-text manuscripts and one abstract reported INR goal achievement data that were included in the meta-analysis [20, 22, 25–28, 30–33]. The defined INR goal for these studies was ≤1.5 (*n* = 5), ≤ 1.4 (*n* = 2), or ≤ 1.3 (*n* = 2). There were 313 of 365 patients who met goal INR in the PCC4 group compared to 360 of 572 patients in the PCC3 group. In the evaluation of achieving goal INR, the calculated ORs from the individual studies ranged from 1.11 to 14.44 in favor of the odds of PCC4 reversing the INR compared to PCC3. The estimated average OR for all included studies based on the REM was 3.61 (95% CI: 1.97–6.60, *p* < 0.001). Among the included studies, research previously done by the authors (DM, SC, JW) had the largest positive association between the effect of PCC4 on INR reversal compared to PCC3 (OR = 14.44, 95% CI: 3.80–54.94) [31]. However, removing this study from the meta-analysis did not have a large effect on the overall meta-analysis findings (OR = 3.17, 95% CI: 1.89–5.33).

**Fig. 2:**
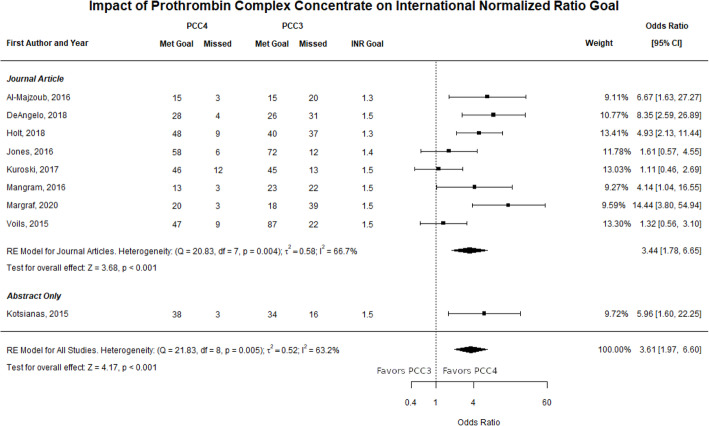
Forest plot INR goal

**Fig. 3:**
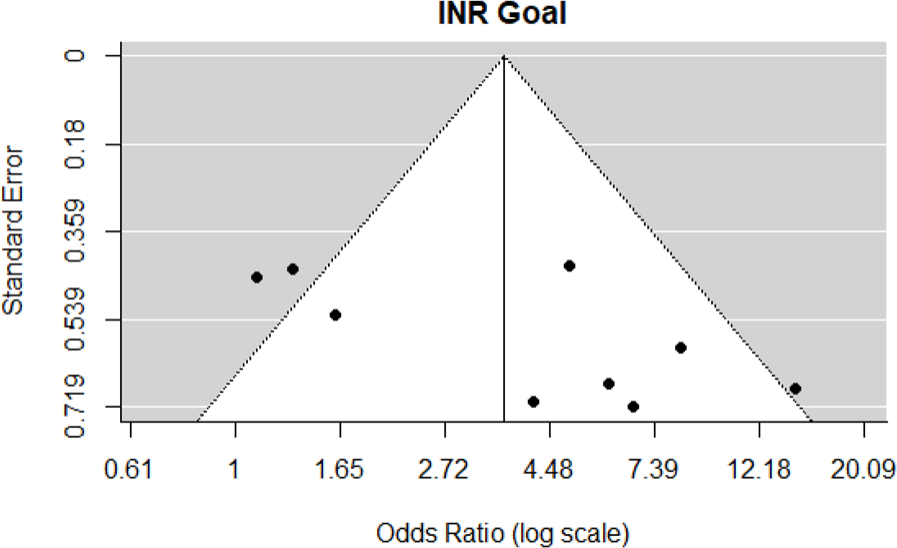
Funnel plot INR goal

Since only one abstract reported group numbers, we performed subgroup analysis excluding the abstract data and found similar results favoring PCC4 over PCC3 odds for achieving goal INR (OR = 3.44, 95% CI: 1.78–6.65, *p* < 0.001). A forest plot showing the observed outcomes and the estimates based on the REMs is shown in Fig. 2. There is significant heterogeneity in the individual study ORs in the meeting goal INR outcome (*Q* = 21.83, *df* = 8, *p =* 0.005, τ^2^ = 0.52, *I*^2^ = 63.2%). A funnel plot of the OR estimates is shown in Fig. 3. The rank correlation and regression test did not indicate funnel plot asymmetry (*p* = 0.060 and *p* = 0.36, respectively).

Three abstracts did not provide the number of patients in each group and therefore were not included in the meta-analysis [21, 29, 34]. They did report the percentage of patients achieving goal INR and associated odds ratios. All three reports favored PCC4 over PCC3. Cang, et al. reported the odds of PCC4 patients achieving the INR goal was eleven times greater than PCC3 patients (OR = 11.3, 95% CI: 3.8–33.9, *p* < 0.001) based on a greater proportion of patients meeting goal INR (PCC4: 74.3% vs. PCC3: 35.2%, no *p*-value reported) [21]. Peck et al. used a multivariable model adjusting for age, sex, body mass index (BMI), and baseline INR to estimate that PCC4 had eighteen times to odds of achieving goal INR goal compared to PCC3 (OR = 18.1, 95% CI: 3.1–106.5) based on the goal INR achievement in the groups (PCC4: 53.8% vs. PCC3: 18.4%, *p* = 0.005) [29]. Wagner et al. reported a greater frequency of achieving an INR < 1.4 in patients given PCC4 than PCC3 (45.2% vs 27.3%, *p* < 0.01) [34]. However, the authors appeared to have calculated the percent based on the total study population rather than group size. If this is the case, the approximate percentage of patients who achieved goal INR was 90% in the PCC4 group and 55% in the PCC3 group.

### Thromboembolic events

**Fig. 4:**
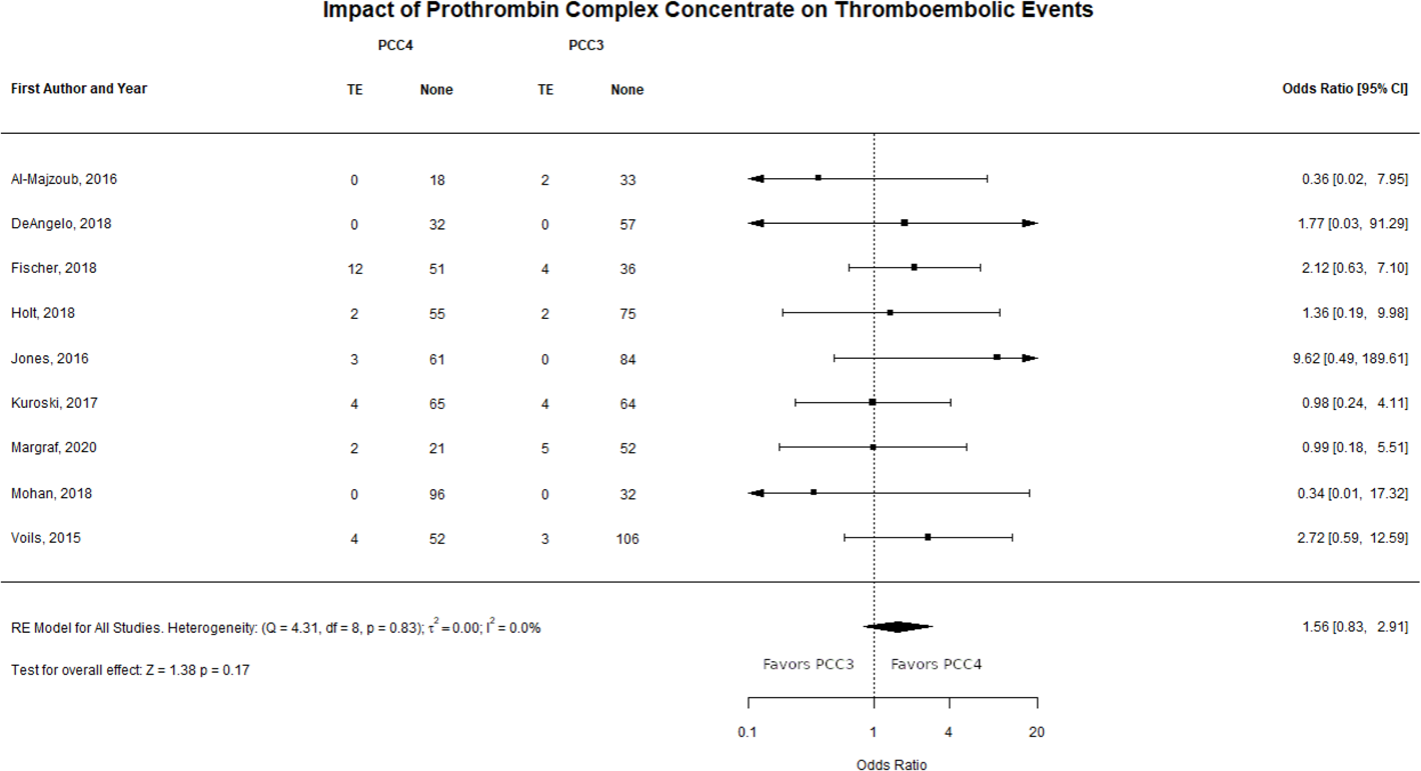
Forest plot for thromboembolic events

Nine journal articles reported TE outcomes included in the meta-analysis [20, 22, 24, 25, 27, 28, 31–33]. There were 27 of 478 patients who had TE in the PCC4 group compared to 20 of 559 patients in the PCC3 group. The observed ORs from these studies ranged from 0.36 to 9.62. The estimated average OR based on the REM was 1.56 (95% CI: 0.83–2.91, *p* = 0.17). This indicates that PCC4 is associated with a 56% increase in TE compared to PCC3; however, this outcome was not statistically significant. A forest plot showing the observed outcomes and the estimates based on the random-effects models is shown in Fig. 4.

**Fig. 5:**
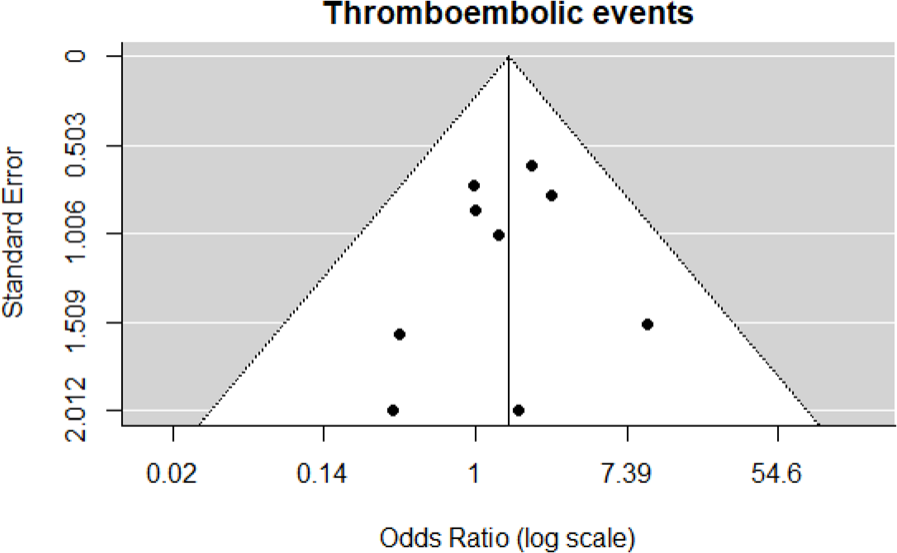
Funnel plot for thromboembolic events

No heterogeneity was found in the TE outcomes (*Q* = 4.31, df = 8, *p* = 0.83, τ^2^ = 0.00, *I*^2^ = 0.00%). A funnel plot of the OR estimates is shown in Fig. 5. The rank correlation and regression test did not indicate funnel plot asymmetry (*p* = 0.76 and *p* = 0.59, respectively). Two studies reported no events in either treatment group [22, 32]. Removing them from the analysis had no major impact on the interpretation of the results (OR = 1.61, 95% CI: 0.85–3.08, *p* = 0.15).

One full-text manuscript reported TE outcomes but was excluded from the TE meta-analysis because it did not separate events in patients on rivaroxaban from those receiving warfarin [30]. One abstract reported the rate of early TE complications as the primary outcome and found this occurred 11.2% in PCC4 patients and 9.6% in PCC3 patients [34]. However, it is unclear if they calculated these percentages based on total study size or group size.

### Survival during hospital stay

**Fig. 6:**
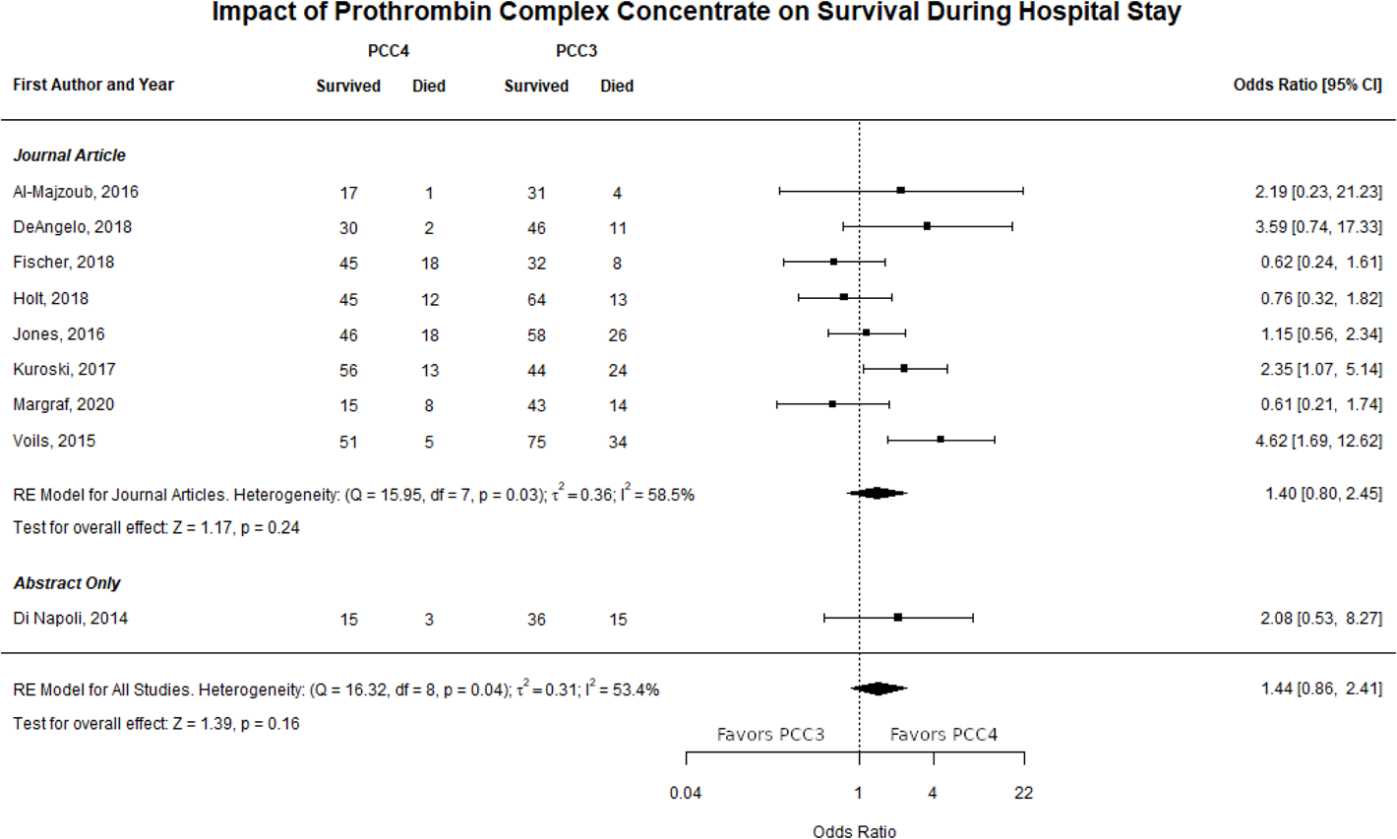
Forest plot survival during hospital stay

**Fig. 7:**
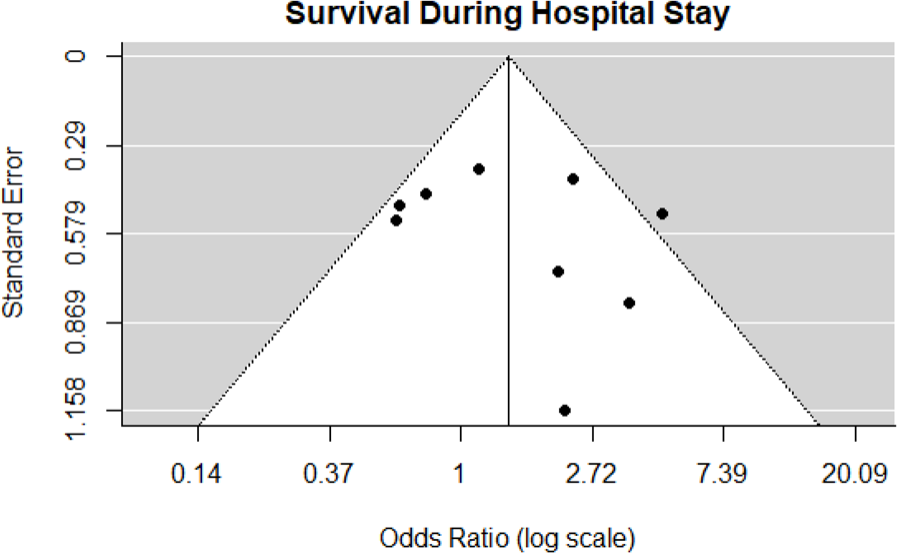
Funnel plot survival during hospital stay

Eight journal articles and one abstract reported survival during hospital stay data that were used in the meta-analysis [20, 22–25, 27, 28, 31, 33]. There were 320 of 400 patients who survived during hospital stay PCC4 group compared to 429 of 578 patients in the PCC3 group. There was a 44% increase in the odds of survival during hospital stay associated with PCC4 compared to PCC3, but this outcome was not statistically significant. The observed ORs from these studies ranged from 0.33 to 4.62, and the estimated average OR based on the REM was 1.44 (95% CI: 0.86–2.41, *p* = 0.16). The subgroup analysis on full-text journal articles was similar, (OR = 1.40, 95% CI: 0.80–2.45, *p* = 0.24). A forest plot showing the observed outcomes and the estimates based on the random-effects models is shown in Fig. 6. A significant amount of heterogeneity was found in the outcomes (*Q* = 16.32, df = 8, *p* = 0.04, τ^2^ = 0.31, *I*^2^ = 53.4%). A funnel plot of the OR estimates is shown in Fig. 7. The rank correlation and regression test did not indicate funnel plot asymmetry (*p* = 0.92 and *p* = 0.40, respectively).

One study reported two deaths in each PCC product group but was excluded from the meta-analysis because it did not separate deaths in patients on rivaroxaban from those taking warfarin [30]. One abstract not included in the meta-analysis reported mortality was lower in the PCC4 group than the PCC3 group, 15.2% versus 26.4%, respectively [34]. It is uncertain if this is based on total or group size.

### Additional analysis

None of the studies in the INR goal, TE, or survival during hospital stay were outliers after examination of the studentized residuals, and none of the studies were overly influential in the model according to Cook’s distances.

Leave-one-out sensitivity analysis did not reveal that any study had a drastic effect on the INR goal outcome. The ORs range from 3.14 to 4.34, as seen in Additional file 1: Appendix C-Table 1; no one study loses significance in favor of PCC4. Similar findings were found with the other outcomes: TE and survival during hospital stay. Odds ratios ranged from 1.24 to 1.56, and 1.14 to 1.49, respectively.

The estimates and intervals from the GLMM analyses were similar to the REM models (Additional file 1: Appendix C – Table 2-3): INR Goal (OR = 3.79, 95% CI: 2.13–6.74); TE (OR = 1.33, 95% CI: 0.75–2.38); survival during hospital stay (OR = 1.39, 95% CI: 0.86–2.25).

## Discussion

Rapid and reliable reversal of the INR in warfarin anticoagulated patients who experience a critical bleed, trauma, or the need for emergent surgery is necessary to provide effective care during these critical events. Pre-reversal INR, the dose of PCC administered, adjunct therapies such as vitamin K and FFP, and the composition of coagulation factors in PCC products can all be factors to consider when evaluating PCC products’ ability to reverse INR. While several small, single-center research comparisons of PCC3 and PCC4 have been published, a large, randomized comparison of the efficacy and safety of these products has not been done. Whether there is a difference in the ability of PCC3 or PCC4 to effectively and safely reverse INR is unknown.

This systematic review and meta-analysis of studies comparing PCC3 and PCC4 for emergent warfarin reversal found the odds of reversal of INR to defined goal INR was more likely with PCC4, and there was little difference in the odds of experiencing a TE or hospital mortality. Patients who received PCC4 for EWR had more than three times the odds of achieving goal INR than patients who received PCC3. There was little difference in the odds of TE or hospital mortality.

This is the first systematic review and meta-analysis of studies directly comparing PCC3 and PCC4 safety and effectiveness for EWR. A previous systematic review without meta-analysis investigated decreasing the INR to ≤1.5 within 1 hour of PCC administration in PCC4 and PCC3, but no direct comparisons between the treatments had been published at the time of their publication [19]. They included eight studies investigating PCC3, ten for PCC4, and found PCC4 was more effective than PCC3 in decreasing the INR to ≤1.5.

Several possible considerations are underlying the findings. First, the compositional differences in the PCC products, specifically concerning the amount of factor VII. Given the INR is most sensitive to factor VII, a lack of replacement of this factor could account for the comparative weaker response to INR lowering with PCC3. Second, PCC4 products were studied in clinical trials for use in warfarin reversal using a predefined weight and INR-based dosing strategy, whereas PCC3 products have been prescribed off-label and with no dosing recommendations, thus leading to prescribing based on unvalidated dosing strategies. Therefore, hospital treatment protocols tend to follow manufacturer dosing guidance for PCC4 whereas PCC3 protocols vary between providers and hospital systems. Third, there is little overlap in the use of PCC3 and PCC4 in the studies. Most institutions treated patients with PCC3 products, the only available products available in the US before the approval of a PCC4 product, and then switched to PCC4 once it became available. As such, there may be a temporal effect in the overall OR that cannot be ruled out.

Studies comparing PCC3 to PCC4 outside of the setting of EWR have similar results. Zeeshan et al. found faster correction of INR with PCC4 compared to PCC3 in a propensity-score-matched analysis of patients treated for coagulopathy of trauma (PCC4: *n* = 125, 365 min vs. PCC3: n = 125, 428 min, *p* < 0.01), and fewer units of FFP transfused (6 units vs. 8 units, *p* < 0.03) [53]. Also, the incidence of TE and mortality were similar between the PCC groups. Although their study excluded patients who were receiving preinjury warfarin anticoagulants and other anticoagulants, the findings suggest the increased effectiveness and reduction of FFP use may be due to the role that factor VII plays in the coagulation cascade. Regardless of prior warfarin treatment, the administration of PCC4 with a higher factor VII concentration leads to faster correction of the INR.

While the focus of this current study investigates non-activated PCC3 and PCC4 products, other blood factor products, not included in this analysis, have been used as hemostatic agents alone or in combination with PCC3, vitamin K, and FFP. Factor eight (VIII) inhibitor bypassing activity (FEIBA®), an activated PCC (aPCC) product, contains mainly non-activated factors II, IX, and X and activated factor VII [12]. Coagulation factor VII activated (NovoSeven® RT) is a single coagulation factor product [13]. These products are approved by the US Food and Drug Administration (FDA) for indications to control and prevent bleeding in hemophilia A and B [12, 13]. Factor VIIa has been used alone and in combination with PCC3 to compensate for the low amount of factor VII [14, 15]. Activated PCC has been used off-label for EWR [54]. Given the differing composition of these products as containing active factor VII and that guidelines do not currently recommend these products for EWR, we chose to not include these products in our analysis.

The clinical implications of our findings serve to reinforce current recommended guidelines; in most cases, PCC4 has surpassed PCC3 for EWR in the US with FFP as a second-line agent. In a recent survey of 281 critical care and emergency medicine pharmacists, 92.9% reported the use of PCC4 for warfarin reversal. However, only 58.7% of them reported the use of the labeled weight-based dosing strategy for this indication. Of those not following the FDA-labeled dose, 30.6% used a fixed-dose regimen, commonly 1500 units once [55]. Since the safety profile is similar between PCC treatments, PCC3 could be used as an alternative for PCC4 when it is unavailable. Guidelines for the management of warfarin-induced intracerebral hemorrhage recommend PCC3 or PCC4 as valid treatment options [2, 7].

Although cost-effectiveness was not a focus of our research, it is important in the overall anticoagulation strategy, drug purchasing, and formulary decisions. Two of the studies in our research addressed this issue. Mangram estimated that cost-effectiveness, as determined by comparing the total cost of all reversal agents used per successful reversal, favored PCC4 ($3797) over PCC3 ($5382) even though PCC4 had a higher initial acquisition cost [30]. DeAngelo found the cost-effective ratio, the total reversal cost divided by the proportion of patients that achieved anticoagulation reversal, favored PCC4 ($5834) over PCC3 ($8033) [22]. As of September 2021, the wholesale acquisition cost listed in RED BOOK for Kcentra® is $2.62 per unit versus Profilnine® at $1.35 per unit for the 500 unit vials of each product [56]. However, the higher acquisition cost of PCC4 may be offset by several factors including reduced dosing requirements and fewer additional reversal agents used to achieve anticoagulation reversal.

There are several limitations to study findings associated with a meta-analysis of observational studies. The selection of a preferred PCC product in the US has been driven by product availability (only PCC3 products before 2013) and FDA approval (PCC4 after 2013) of PCC4 after clinical trial results demonstrating efficacy over FFP. While both PCC3 and PCC4 products continue to be available for clinicians to prescribe for EWR, clinical evaluation of the effectiveness and safety comparing PCC4 and PCC3 products has not been the guiding principle for their clinical use.

None of the studies included in this systematic review and meta-analysis were randomized controlled trials, which would limit bias in PCC treatment effect findings by design, and where all patients would follow the same protocol. An inherent difficulty with retrospective cohort analysis is few study-level variables or factors are the same for all patients. In the critical care setting, an RCT study design is difficult to implement. So, a reasonable alternative given the constraints is to retrieve electronic health records collected during clinical care to estimate the difference in effectiveness and safety between PCC3 and PCC4 treatment protocols.

Answering clinical questions is limited by the variety of reporting methods authors chose. For example, change in INR after PCC administration is an outcome of interest to clinicians as a direct measure of treatment effect. However, there is no reliable method to combine these two measures of central tendency into a meta-analytic model without introducing bias [38, 57]. Additionally, there is an inherent difficulty in estimating the treatment effect on INR reduction attributed to one treatment when several concomitant therapies which also reduce INR are given. There is no way to determine the effect of any one of these factors with the variability at which they happen during clinical care with an observational, retrospective study design.

An additional limitation is the heterogeneity of the studies included in the analysis, resulting from the various treatment protocols, the patient populations, and study variations in the literature. Despite these limitations, this research has added an understanding of the effectiveness and safety profiles of PCC products used during clinical care.

## Conclusion

This systematic review and meta-analysis has gathered and reviewed the current research regarding direct comparisons of PCC4 and PCC3. The qualitative, and quantitative analyses provide OR estimates of the differences in effectiveness and safety of PCC4 compared to PCC3. There are greater odds of achieving INR reversal to goal INR in the setting of emergent warfarin reversal with PCC4 versus PCC3. However, thromboembolic events, and survival during patient hospital stay are similar between the PCC products.

Future research should investigate the effect of PCC4 in terms of reduction of INR and thrombin generation assays to evaluate for hemostasis. Ideally, this will require carefully timed discontinuation of warfarin, administration of PCC4, and accurate measurements, which are factors difficult to control when treating critically ill patients.

## Supplementary Information

**Additional file 1.**

## Data Availability

The datasets used and/or analyzed during the current study are available from the corresponding author on reasonable request.

## Abbreviations

CI: Confidence interval
EWR: Emergent warfarin reversal
FDA: Food and Drug Administration
FFP: Fresh frozen plasma
GI: Gastrointestinal
GLMM: Generalized linear mixed model
ICH: Intracerebral hemorrhage
INR: International normalized ratio
IRB: Institutional review board
ISI: International sensitivity index
OR: Odds ratio
PCC: Prothrombin complex concentrate
PCC3: Three-factor prothrombin complex concentrate
PCC4: Four-factor prothrombin complex concentrate
RCT: Randomized controlled trial
rFVIIa: Recombinant factor VII activated
TE: Thromboembolic events
