# Supplementary material for: Comparison of 3-factor versus 4-factor prothrombin complex concentrate for emergent warfarin reversal: a systematic review and meta-analysis
Source: BMC Emerg Med. 2022 Jan 24;22:14. doi: 10.1186/s12873-022-00568-x (PMC8785536; doi:10.1186/s12873-022-00568-x)
Supplement: Supplementary file 1 — Additional file 1. [file 12873_2022_568_MOESM1_ESM.docx]

# **Appendix A – Search Strategy**

**Ovid MEDLINE**

Ovid MEDLINE(R) and Epub Ahead of Print, In-Process & Other Non-Indexed Citations, Daily and Versions(R)

1. ("prothrombin complex concentrate*" or PCC*).tw.
2. (3-Factor or three-factor or 3F-PCC or PCC3).tw.
3. (preconativ or konyne or factorIXa or prothrombinex or bebulin or profilnine or cofact).tw.
4. 2 or 3
5. (4-factor or four-factor or 4F-PCC or PCC4).tw.
6. (beriplex or prothromplex or proplex or octaplex or PPSB-HT or kcentra).tw.
7. 5 or 6
8. ((warfarin or vitamin K antagonist) adj3 revers*).tw.
9. exp Factor IX/
10. exp Anticoagulants/pd, tu [Pharmacology, Therapeutic Use]
11. complex.tw,kf.
12. factor IX complex.tw.
13. 4 and 7
14. 9 and 10 and 11
15. 1 and 8
16. 1 and 13
17. 12 or 14 or 15 or 16

**Embase**

Embase+Embase Classic

1. exp prothrombin complex/
2. ("prothrombin complex concentrate*" or PCC*).tw.
3. 1 or 2
4. (3-Factor or three-factor or 3F-PCC or PCC3).tw.
5. (preconativ or konyne or factorIXa or prothrombinex or bebulin or profilnine or cofact).tw.
6. 4 or 5
7. (4-factor or four-factor or 4F-PCC or PCC4).tw.
8. (beriplex or prothromplex or proplex or octaplex or PPSB-HT or kcentra).tw.
9. 7 or 8
10. ((warfarin or vitamin K antagonist) adj3 revers*).tw.
11. exp blood clotting factor 9/
12. anticoagulant agent/
13. complex.tw,kw.
14. factor ix complex.tw.
15. 6 and 9
16. 11 and 12 and 13
17. 3 and 10
18. 3 and 15
19. 14 or 16 or 17 or 18

**Cochrane Library**

1. ("prothrombin complex concentrate*" or PCC*):ti,ab,kw
2. (3?Factor or "three?factor" or 3F?PCC or PCC3):ti,ab,kw
3. (preconativ or konyne or factorIXa or prothrombinex or bebulin or profilnine or cofact):ti,ab,kw
4. #2 or #3
5. (4?factor or "four?factor" or 4F?PCC or PCC4):ti,ab,kw
6. (beriplex or prothromplex or proplex or octaplex or PPSB-HT or kcentra):ti,ab,kw
7. #5 or #6
8. ((warfarin or vitamin K antagonist) Near/3 revers*):ti,ab,kw
9. MeSH descriptor: [Factor IX] explode all trees
10. MeSH descriptor: [Anticoagulants] explode all trees
11. (complex):ti,ab,kw
12. (factor ix complex):ti,ab,kw
13. #4 and #7
14. #9 and #10 and #11
15. #1 and #8
16. #1 and #13
17. #12 or #14 or #15 or #16

**Scopus**

( TITLE-ABS-KEY ( "prothrombin complex concentrate*" OR pcc ) AND ( TITLE-ABS-KEY ( warfarin OR ( "vitamin K antagonist" W/3 revers* ) OR ( ( "3-Factor" OR "three-factor" OR "3F-PCC" OR pcc3 OR preconativ OR konyne OR factorixa OR prothrombinex OR bebulin OR profilnine OR cofact ) AND ( "4-factor" OR "four-factor" OR "4F-PCC" OR pcc4 OR beriplex OR prothromplex OR proplex OR octaplex OR ppsb-ht OR kcentra ) ) ) ) ) OR TITLE-ABS ( "factor ix complex" OR "factor 9 complex" )

**Appendix B: Data extraction form**

**Questions in the Newcastle-Ottawa quality assessment score form:**

Representativeness of the PCC4 cohort

Selection of the PCC3 cohort

Ascertainment of exposure

Demonstration that the outcome of interest was not present at the start of study

Study controls for time related to PCC dose and/or INR

Study controls for any additional factor

Assessment of outcome

Were all patients measured for both initial and follow-up INR? (Follow-up)

Adequacy of follow-up of cohorts

**Data extraction form questions:**

Other non-warfarin patients

Other reported post-PCC INR goal

INR change PCC4 group

Subgroup result PCC4 group

Thromboembolic events in PCC4 group

Survival/Death in the PCC4 group

Goal INR achieved PCC4 group

Age for PCC4 group

Weight for PCC4 group

Sex for PCC4 group

Proportion of GI Bleeds for PCC4 group

Proportion of ICH for PCC4 group

Proportion of bleed types listed as "Other" PCC4 group

Proportion of bleed types not listed above PCC4 group

Number or percentage of FFP units used in PCC4 group

Vitamin K usage in the PCC4 group

**Appendix C: Supplemental Tables**

**Table 1.** Leave-one-out sensitivity analysis from random-effects models.

| **Study** | **Outcome** | **OR estimate** | **95% CI** | **P-value** |
| --- | --- | --- | --- | --- |
| Al-Majzoub, 2016 | INR Goal | 3.48 | (1.81-6.70) | <0.001 |
| DeAngelo, 2018 | INR Goal | 3.33 | (1.76-6.32) | <0.001 |
| Holt, 2018 | INR Goal | 3.56 | (1.78-7.10) | <0.001 |
| Jones, 2016 | INR Goal | 4.12 | (2.14-7.93) | <0.001 |
| Kuroski, 2017 | INR Goal | 4.34 | (2.39-7.88) | <0.001 |
| Mangram, 2016 | INR Goal | 3.59 | (1.84-7.00) | <0.001 |
| Margraf, 2020 | INR Goal | 3.14 | (1.75-5.64) | <0.001 |
| Voils, 2015 | INR Goal | 4.27 | (2.29-7.99) | <0.001 |
| Kotsianas, 2015 | INR Goal | 3.51 | (1.81-6.82) | <0.001 |
| Al-Majzoub, 2016 | TE | 1.66 | (0.87-3.14) | 0.12 |
| DeAngelo, 2018 | TE | 1.55 | (0.82-2.93) | 0.18 |
| Fischer, 2018 | TE | 1.39 | (0.67-2.89) | 0.38 |
| Holt, 2018 | TE | 1.58 | (0.81-3.06) | 0.18 |
| Jones, 2016 | TE | 1.43 | (0.75-2.72) | 0.28 |
| Kuroski, 2017 | TE | 1.74 | (0.86-3.49) | 0.12 |
| Margraf, 2020 | TE | 1.67 | (0.85-3.27) | 0.14 |
| Mohan, 2018 | TE | 1.62 | (0.86-3.06) | 0.14 |
| Voils, 2015 | TE | 1.39 | (0.70-2.77) | 0.35 |
| Al-Majzoub, 2016 | Survival | 1.32 | (0.78-2.23) | 0.31 |
| DeAngelo, 2018 | Survival | 1.25 | (0.74-2.11) | 0.4 |
| Fischer, 2018 | Survival | 1.49 | (0.88-2.54) | 0.14 |
| Holt, 2018 | Survival | 1.46 | (0.84-2.54) | 0.18 |
| Jones, 2016 | Survival | 1.38 | (0.77-2.50) | 0.28 |
| Kuroski, 2017 | Survival | 1.23 | (0.71-2.14) | 0.46 |
| Mangram, 2016 | Survival | 1.44 | (0.86-2.41) | 0.16 |
| Margraf, 2020 | Survival | 1.48 | (0.87-2.52) | 0.15 |
| Voils, 2015 | Survival | 1.14 | (0.73-1.77) | 0.56 |
| Di Napoli, 2014 | Survival | 1.29 | (0.75-2.24) | 0.36 |

**Table 2.** Results from estimation from generalized linear mixed-effects models.

| **Outcome** | **OR estimate** | **95% CI** | **P-value** |
| --- | --- | --- | --- |
| INR Goal | 3.79 | (2.13-6.74) | <0.001 |
| TE | 1.66 | (0.89-3.07) | 0.11 |
| Survival | 1.39 | (0.86-2.25) | 0.17 |

**Table 3.** Leave-one-out sensitivity analysis from generalized linear mixed-effects models.

| **Study** | **Outcome** | **OR estimate** | **95% CI** | **P-value** |
| --- | --- | --- | --- | --- |
| Al-Majzoub, 2016 | INR Goal | 3.59 | (1.93-6.66) | <0.001 |
| DeAngelo, 2018 | INR Goal | 3.43 | (1.87-6.28) | <0.001 |
| Holt, 2018 | INR Goal | 3.67 | (1.90-7.08) | <0.001 |
| Jones, 2016 | INR Goal | 4.22 | (2.27-7.84) | <0.001 |
| Kuroski, 2017 | INR Goal | 4.47 | (2.56-7.81) | <0.001 |
| Mangram, 2016 | INR Goal | 3.70 | (1.97-6.94) | <0.001 |
| Margraf, 2020 | INR Goal | 3.21 | (1.85-5.57) | <0.001 |
| Voils, 2015 | INR Goal | 4.39 | (2.43-7.91) | <0.001 |
| Kotsianas, 2015 | INR Goal | 3.61 | (1.93-6.75) | <0.001 |
| Al-Majzoub, 2016 | TE | 1.82 | (0.96-3.45) | 0.07 |
| Fischer, 2018 | TE | 1.51 | (0.72-3.15) | 0.27 |
| Holt, 2018 | TE | 1.69 | (0.88-3.25) | 0.11 |
| Jones, 2016 | TE | 1.44 | (0.76-2.72) | 0.27 |
| Kuroski, 2017 | TE | 1.86 | (0.94-3.70) | 0.08 |
| Margraf, 2020 | TE | 1.81 | (0.92-3.55) | 0.09 |
| Voils, 2015 | TE | 1.50 | (0.77-2.95) | 0.24 |
| Al-Majzoub, 2016 | Survival | 1.40 | (1.02-1.92) | 0.04 |
| DeAngelo, 2018 | Survival | 1.29 | (0.79-2.12) | 0.31 |
| Fischer, 2018 | Survival | 1.56 | (0.95-2.55) | 0.08 |
| Holt, 2018 | Survival | 1.52 | (0.90-2.55) | 0.11 |
| Jones, 2016 | Survival | 1.44 | (0.82-2.51) | 0.20 |
| Kuroski, 2017 | Survival | 1.28 | (0.76-2.17) | 0.36 |
| Mangram, 2016 | Survival | 1.48 | (0.91-2.40) | 0.11 |
| Margraf, 2020 | Survival | 1.53 | (0.93-2.52) | 0.09 |
| Voils, 2015 | Survival | 1.18 | (0.79-1.76) | 0.43 |
| Di Napoli, 2014 | Survival | 1.34 | (0.80-2.25) | 0.27 |
